# Identification of New miRNA-mRNA Networks in the Development of Non-syndromic Cleft Lip With or Without Cleft Palate

**DOI:** 10.3389/fcell.2021.631057

**Published:** 2021-03-01

**Authors:** Chengyi Fu, Shu Lou, Guirong Zhu, Liwen Fan, Xin Yu, Weihao Zhu, Lan Ma, Lin Wang, Yongchu Pan

**Affiliations:** ^1^Jiangsu Key Laboratory of Oral Diseases, Nanjing Medical University, Nanjing, China; ^2^Department of Orthodontics, Affiliated Stomatological Hospital, Nanjing Medical University, Nanjing, China; ^3^State Key Laboratory of Reproductive Medicine, Nanjing Medical University, Nanjing, China

**Keywords:** non-syndromic cleft lip with or without cleft palate, let-7c-5p, PIGA, TGFB2, MiR-193a-3p

## Abstract

**Objective:** To identify new microRNA (miRNA)-mRNA networks in non-syndromic cleft lip with or without cleft palate (NSCL/P).

**Materials and Methods:** Overlapping differentially expressed miRNAs (DEMs) were selected from cleft palate patients (GSE47939) and murine embryonic orofacial tissues (GSE20880). Next, the target genes of DEMs were predicted by Targetscan, miRDB, and FUNRICH, and further filtered through differentially expressed genes (DEGs) from NSCL/P patients and controls (GSE42589), MGI, MalaCards, and DECIPHER databases. The results were then confirmed by *in vitro* experiments. NSCL/P lip tissues were obtained to explore the expression of miRNAs and their target genes.

**Results:** Let-7c-5p and miR-193a-3p were identified as DEMs, and their overexpression inhibited cell proliferation and promoted cell apoptosis. *PIGA* and *TGFB2* were confirmed as targets of let-7c-5p and miR-193a-3p, respectively, and were involved in craniofacial development in mice. Negative correlation between miRNA and mRNA expression was detected in the NSCL/P lip tissues. They were also associated with the occurrence of NSCL/P based on the MGI, MalaCards, and DECIPHER databases.

**Conclusions:** Let-7c-5p-*PIGA* and miR-193a-3p-*TGFB2* networks may be involved in the development of NSCL/P.

## Introduction

Non-syndromic cleft lip with or without cleft palate (NSCL/P) is one of the most common congenital craniofacial deformities in humans, which occurs in ~1 in 700 live births worldwide (Birnbaum et al., 2009). It results from defects in normal craniofacial developmental processes during embryonic development (Panamonta et al., 2015; Tillman et al., 2018).

The etiology of NSCL/P is complicated, with the involvement of various genetic and environmental factors. For example, *IRF6, FOXE1, MSX1*, and *BCL3* have been found to be associated with oral cleft (Dixon et al., 2011; Simioni et al., 2015), and environmental factors such as maternal smoking and maternal alcohol during pregnancy also increase the incidence rate of NSCL/P (Molina-Solana et al., 2013). In terms of growth and development, it is often caused by disturbances in the proliferation, migration, and survival of neural crest cells during embryonic development (Lan et al., 2015).

MicroRNAs (miRNAs) are a class of small RNAs originally transcribed from non-coding regions, which are 20 to 24 nucleotides in length. Since their discovery in 1993, miRNAs have been demonstrated to play critical roles in the post-transcriptional regulation of gene expression (Bartel, 2018; Dexheimer and Cochella, 2020). It is known that miRNAs negatively regulate gene expression by binding to complementary sequences in the 3' untranslated region (UTR) of target mRNAs, which can result in the degradation of the mRNA transcript or suppression of the protein translation process (Bartel, 2009). It is estimated that ~60% of human protein-coding genes could be modulated by miRNAs (Richbourg et al., 2020).

Accumulating evidence suggests that miRNA-mRNA networks are involved in craniofacial development. For example, Ling Li found that the negative feedback loop between E2F1 and miR-17-92 may contribute to palatal development by regulating the proliferation and cell cycle of palatal mesenchymal cells (Li et al., 2017). It has been reported that mutation of miR-17-92 often leads to a severe craniofacial phenotype by targeting the Bmp/AP-2α-miR-17-92*-Tbx* pathway (Wang et al., 2013). Our previous study found that the miR-146a rs2910164 G allele was associated with the expression of miR-146a, which contributed to the occurrence of oral cleft by regulating *TRAF6* expression (Pan et al., 2018).

In this study, we synthetically analyzed multiple databases to establish a novel regulatory network of miRNA-mRNA and conducted a series of experiments to verify the function of miRNAs and their target genes.

## Materials and Methods

### Collection of Microarray Datasets

Three datasets of miRNA and gene expression profiles (GSE47939, GSE20880, GSE42589) were obtained from the Gene Expression Omnibus (GEO) database (https://www.ncbi.nlm.nih.gov/geo/) (Edgar et al., 2002). The process for filtering the datasets was applied as follows. First, we searched for NSCL/P-related miRNAs in the GEO database using the keywords “cleft miRNA” and “orofacial miRNA.” After filtering out irrelevant datasets, GSE47939 and GSE20880 remained. Similarly, we performed another search using the key word “cleft gene.” Considering that only GSE42589 contains samples of NSCL/P patients and controls at the same time, and can be found in PubMed, we finally chose it for our subsequent study.

GSE47939 was downloaded from GPL11487 (Agilent-021827 Human miRNA Microarray) and included 10 palate tissues from non-syndromic cleft palate patients and six from healthy controls. GSE20880 was downloaded from GPL10179 (Miltenyi Biotec miRXplore miRNA Microarray) and contained three murine embryonic orofacial tissues from gestational days (GD) 12, 13, and 14. GSE42589 was downloaded from GPL6244 ([HuGene-1_0-st] Affymetrix Human Gene 1.0 ST Array) and included seven NSCL/P samples and six control samples. Details of each dataset, including the sample descriptions, are provided in Supplementary Table 1.

### Data Processing

The GEO2R online analysis tool based on GEOquery and limma R package was used to identify differentially expressed miRNAs (DEMs) and differentially expressed genes (DEGs) between groups in a linear model (Ritchie et al., 2015).

A *P* < 0.05 and |logFC| > 1 were used as cut-off criteria for screening DEMs and DEGs. Given that the two miRNA datasets come from different species, miRBase, which assigns the same number to homologous miRNA loci in different species, was used to identify human miRNAs that are homologous to mouse miRNAs (Kozomara and Griffiths-Jones, 2014; Li et al., 2020). Venn diagrams were drawn using the FUNRICH software (Pathan et al., 2015).

### Prediction and Bioinformatic Analysis of miRNA Target Genes

We predicted the miRNA target genes using the TargetScan, miRDB, and FUNRICH software (http://www.targetscan.org/vert_72/, http://mirdb.org/, http://www.funrich.org). MGI (http://www.informatics.jax.org/) (Krupke et al., 2017; Smith et al., 2019), MalaCards (https://www.malacards.org/) (Rappaport et al., 2017), and DECIPHER (https://decipher.sanger.ac.uk/browser) (Firth et al., 2009) were used to annotate the function of target genes related to the development of NSCL/P. The MGI database contains integrated genetic, genomic, and biological data aimed at facilitating the study of human health and disease. The MalaCards human disease database is an integrated database of human maladies and their annotations. The DECIPHER database is an accessible online repository of genetic variation with associated phenotypes that facilitates the identification and interpretation of pathogenic genetic variation in patients with rare disorders.

### Cell Culture and Clone Construction

Human embryonic palatal mesenchyme (HEPM) cells were cultured in Eagle's minimum essential medium containing 10% fetal bovine serum (FBS) and 1% penicillin–streptomycin solution in 5% CO_2_ at 37°C, while human oral epithelial cells (HOECs) were cultured in high-glucose Dulbecco's modified Eagle's medium supplemented with 10% FBS and 1% penicillin–streptomycin solution in 5% CO_2_ at 37°C.

All miRNA mimics were synthesized by GenePharma (Shanghai, China). The *PIGA/TGFB2* 3′-UTR wild type or mutant type fragment was inserted at the Nhel-Xhol restriction site downstream of the luciferase gene in the pmirGLO vector by Promega (Supplementary Figure 1).

### Luciferase Reporter Assays

For the two candidate genes in the 3′-UTR luciferase assay, we transfected the 3′-UTR luciferase reporter plasmids or the corresponding plasmid with mutations and miRNA mimics into HEPM cells and HOECs in 24-well plates using Lipofectamine 2000 (Invitrogen, Carlsbad, CA, USA). After 48 h, firefly luciferase activity was quantified using a luciferase reporter assay system (Promega, Madison, Wisconsin, USA). The ratio of firefly luciferase to Renilla luciferase activity was also assessed. Transfection experiments were performed in triplicates.

### RNA Extraction and Quantitative RT-PCR

Following miRNAs transfection for 48 h, total RNA was extracted from HEPM and HOEC using BioTeke RNApure High-purity Total RNA Rapid Extraction Kit (BioTeke, China) according to the manufacturer's protocols. Quantitative real-time PCR primers were designed and synthesized by TSINGKE (Beijing, China) (Supplementary Table 2). The total RNA was converted into cDNA by using the Reverse Transcription System (ThermoFisher, USA). The relative mRNA expression level and the internal control GAPDH were quantified on ABI PrismR 7900HT Real-Time PCR System (Applied Biosystems). All reactions were conducted in triplicate, and the data were analyzed by the 2–ΔΔCt method (Hu et al., 2016).

### Western Blot Analysis

After transfected with miRNA mimics for 48 h, cellular proteins were extracted using radioimmunoprecipitation (RIPA) buffer (ComWin, Changzhou, China). Protein samples were separated by SDS-PAGE and electrophoretically transferred into polyvinylidene fluoride (PVDF) membranes. The membranes were blocked with 5% fat-free milk for 2 h at room temperature and blotted overnight at 4°C with diluted primary antibody against *PIGA* (phosphatidylinositol glycan anchor biosynthesis class A) (dilution 1:600, ab69768, abcam) or *TGFB2* (transforming growth factor beta 2) (dilution 1:600, cat. no. 1999-1-AP; Proteintech) and GAPDH (dilution 1:1,000, Beyotime, AG019). The proteins in the blot were visualized using ECL (Millipore) and were detected using the Phototope-HRP Western Blot Detection System (Cell Signaling Technology). The observed protein levels were normalized to GAPDH levels.

### Gene Expression During Mouse Craniofacial Development and in Human Lip Samples

In this study, gene expression was assessed in mouse embryonic craniofacial tissues (http://www.facebase.org/, GSE67985) and NSCL/P lip tissue samples.

Gene expression during growth and fusion of the facial prominences in the C57BL/6J mouse strain during embryonic days (E) 10.5–14.5 (GSE67985) were downloaded from GPL1261 ([Mouse430_2] Affymetrix Mouse Genome 430 2.0 Array) in GEO database and contained 60 samples from mice developing maxillary and mandibular processes (Feng et al., 2009).

A total of 40 redundant NSCL/P lip tissues were obtained during surgery, which was approved by the Institutional Review Board of Nanjing Medical University (NJMUERC [2008] No. 20). Informed consent was obtained from all the individuals. Total RNA was isolated using TRIzol reagent (Invitrogen, Carlsbad, CA, USA). The RNA quantity and quality were determined by Nanodrop and 1% agarose electrophoresis. RNA-seq was subsequently performed using Nova6000 (Illumina, CA, USA) by Genergy Bio (Shanghai, China). The average fragments per kilobase of exon model per million fragments mapped (FPKM) value of all samples was used to normalize mRNA expression.

### Collecting Human Subjects, DNA Extraction, Genotyping, and Quality Control

As shown in Supplementary Table 3 and previously reported, genome-wide association studies (GWAS) data consisted of 504 NSCL/P cases and 455 newborn controls were recruited. Venous blood samples were collected according to the protocol of the TIANamp Genomic DNA Kit (TIANGEN, Beijing) from all subjects for genetic analysis. Genotyped were performed using Affymetrix Axiom Genome-Wide CHB1 and CHB2 by CapitalBio Corporation (1,280,786 single nucleotide polymorphisms, SNPs). Sex chromosomes were not included in the genotyping array (Sun et al., 2015). Systematic quality control was performed to filter single nucleotide polymorphisms (SNPs), including Hardy–Weinberg equilibrium ≥ 0.05, minor allele frequency ≥ 5%, and call rate ≥ 95%.

### Functional Annotation of Single-Nucleotide Polymorphisms

HaploReg and 3DSNP were used to annotate the functions of the SNPs. HaploReg (www.broadinstitute.org/mammals/haploreg/haploreg.php) (Ward and Kellis, 2016) is a tool for exploring annotations of the non-coding genome at variants on haplotype blocks, such as candidate regulatory SNPs at disease-associated loci. Three-dimensional (3D) chromatin looping data (3DSNP, http://biotech.bmi.ac.cn/3dsnp/) (Lu et al., 2017) was used to link promising SNPs to their three-dimensional interacting genes.

### Cell Proliferation and Apoptosis Assays

Human miRNA mimics or non-coding (NC) mimics were transfected into HEPM cells and HOECs in 96-well plates according to the manufacturer's instructions. A total of 10 μl CCK-8 (Dojindo Laboratories, Kumamoto, Japan) was added to each well containing 100 μl culture medium at different time points (0, 24, 48, and 72 h) after transfection. The cells were further incubated at 37°C in 5% CO_2_ for 1 h. The absorbance values were subsequently measured at 450 nm using a SpectraMax 190 spectrophotometer (Molecular Devices, CA, USA).

To analyze the effect of the miRNAs on cell apoptosis, HEPM cells and HOECs cultured in six-well plates were transfected with miRNA mimics or NC mimics. After 48 h, the cells were collected and stained with the Annexin V-FITC/propidium iodide kit (KeyGEN Biotech, Nanjing, China). The cells were incubated in the dark at 20–25°C for 10 min. Apoptotic cells were analyzed using flow cytometry and FlowJo™ v10 software (FlowJo LLC, OR, USA).

### Statistical Analysis

To explore DEMs/DEGs in GEO datasets, GEO2R were used with limma R package in a liner model. To evaluate the association between NSCL/P risk and genetic variants, logistic regression analysis under an additive model with adjustment for gender was implemented to calculate the crude and adjusted odds ratios (ORs) and their 95% confidence intervals (CIs). Hardy–Weinberg equilibrium was evaluated in the controls using a goodness-of-fit χ^2^ test. All data were expressed as mean ± standard deviation (SD). Graphs were drawn by GraphPad Prism 8. Differences between groups were analyzed using two-tailed Student's *t*-test. Before *t*-test, normal distribution of all the data were checked using normality test and equality of variances were checked using F-test. A *P* < 0.05 was considered statistically significant. All statistical analyses were two-sided and were performed using PLINK 1.07 software or BM SPSS Statistics 22 (Purcell et al., 2007).

## Results

### Identification of Differentially Expressed miRNAs Associated With Non-syndromic Cleft Lip With or Without Cleft Palate

**Figure 1:**
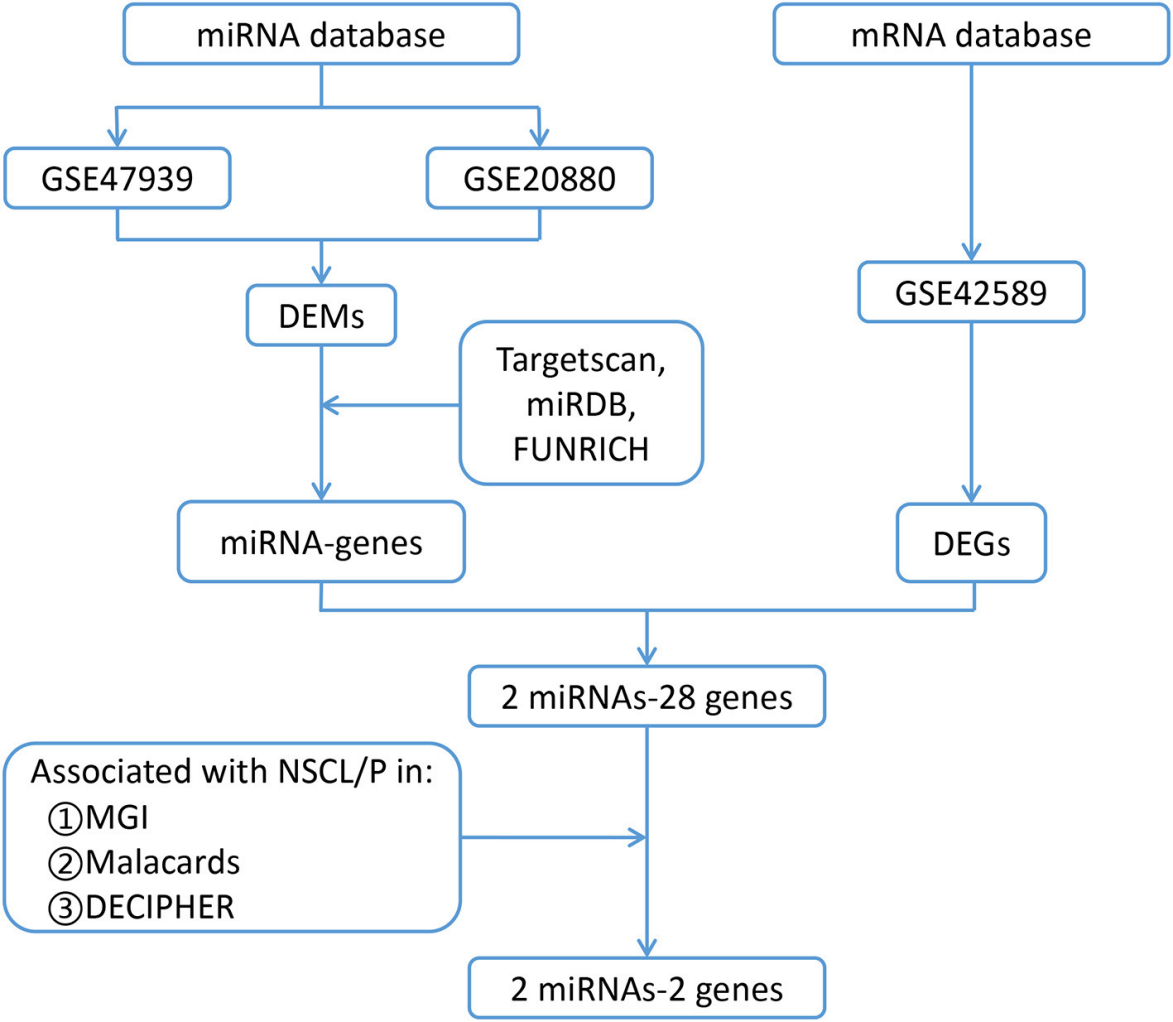
Flow chart for identification of the two miRNAs and their two target genes that are associated with non-syndromic orofacial clefts. DEMs, differentially expressed microRNAs; DEGs, differentially expressed genes; NSCL*/*P, Non-syndromic cleft lip with or without palate; GSE47939, contained 10 non-syndromic cleft palate patients and six controls; GSE20880, three murine embryonic orofacial tissues on gestational days 12–14; GSE42589, included seven NSCL/P samples and six control samples; MGI, Mouse Genome Informatics database; MalaCards, A Comprehensive Automatically-Mined Database of Human Diseases; DECIPHER, DatabasE of genomiC varIation and Phenotype in Humans using Ensembl Resources, Targetscan, predicts biological targets of miRNAs by searching for the presence of conserved 8mer, 7mer, and 6mer sites that match the seed region of each miRNA (http://www.targetscan.org/vert_72/); miRDB, MicroRNA Target Prediction Database (http://mirdb.org/); FUNRICH, Functional Enrichment analysis tool (http://www.funrich.org software).

**Figure 2:**
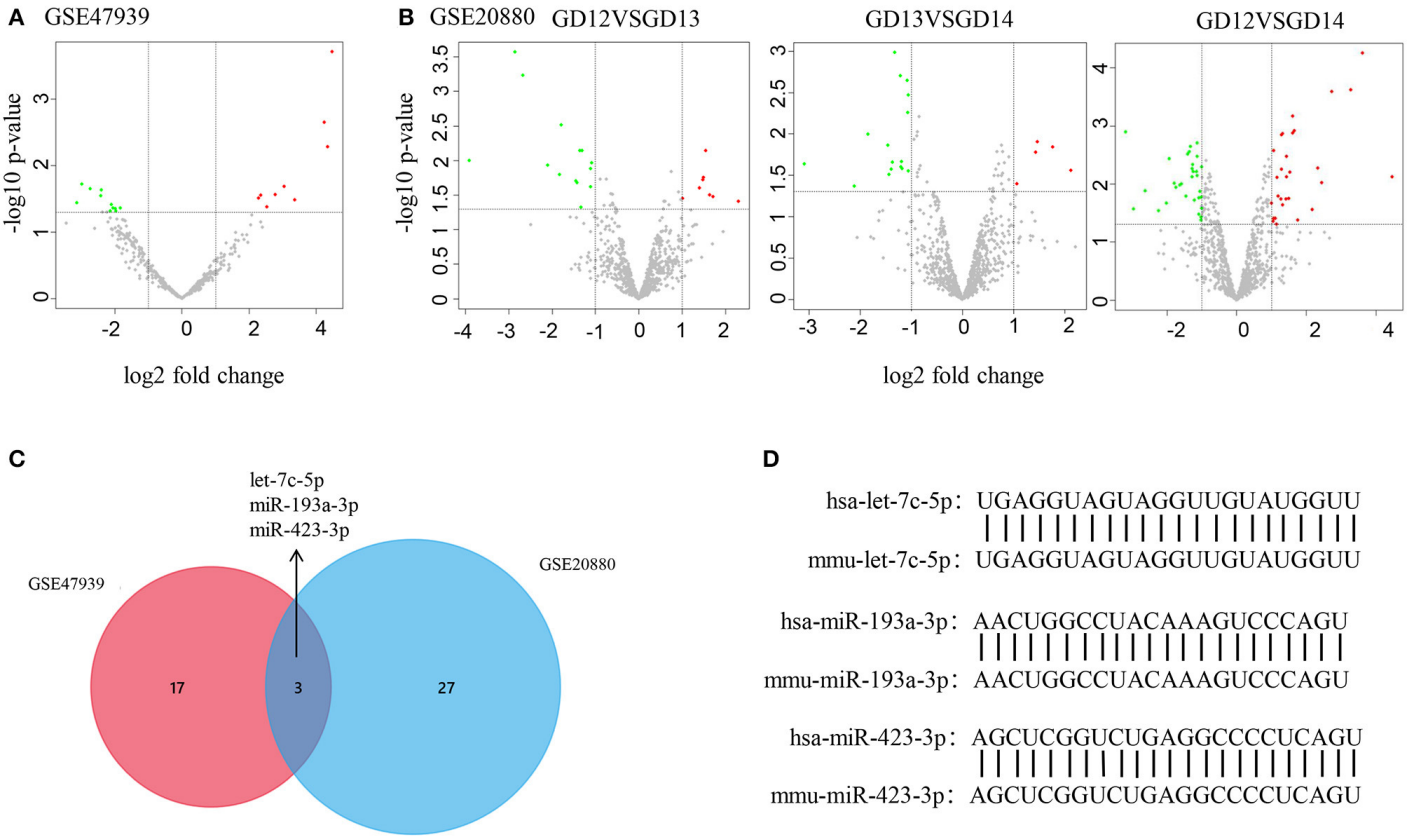
DEM identification by bioinformatics |analysis. **(A,B)** Volcano plot shows DEMs in GSE47939 and GSE20880. Red, green, and gray color indicates relatively high, low, and equal expression of miRNAs, respectively. GD, gestational days. **(C)** Venn diagram shows the overlapping DEMs in GSE47939 and GSE20880. **(D)** Nucleotide sequence comparison between humans and mice for the three overlapping DEMs.

The processes used for screening NSCL/P-associated miRNAs and mRNAs are shown in Figure 1. In this study, first we separately identified the statistically significant DEMs in two miRNA microarray datasets (GSE47939, GSE20880) by GEO2R software, in which the data were all normalized before submitting to GEO database. Next, by combining these statistically significant DEMs in the two miRNA microarray datasets, a total of 47 DEMs were screened out (Supplementary Table 4). Volcano plots were generated to show miRNAs upregulated and downregulated between the two groups (Figures 2A,B). Three overlapping DEMs (let-7c-5p, miR-193a-3p, and miR-423-3p) were identified (Figure 2C). We found that all three miRNAs have conserved sequences in both humans and mice according to miRBase (Figure 2D). The details of these three miRNAs are listed in Table 1.

**Table 1:** LogFC and *P-*values of differentially expressed miRNAs (DEMs) selected from two databases.

	**GSE47939**		**GSE20880**		
**ID**	**LogFC**	***P* value**	**LogFC**	**Gestational days**	***P*-value**
hsa-let-7c-5p	3.13	3.60E-02	−1.07	12VS14	2.68E-03
hsa-miR-193a-3p	−4.26	2.21E-03	−2.85	12VS13	2.71E-04
			−3.62	12VS14	5.60E-05
hsa-miR-423-3p	2.14	4.86E-02	1.58	12VS14	1.01E-02

*DEMs, differentially expressed miRNAs; logFC, log^2^ fold change. P < 0.05 was considered statistically significant*.

### Selection of Potential miRNA Target Genes

**Figure 3:**
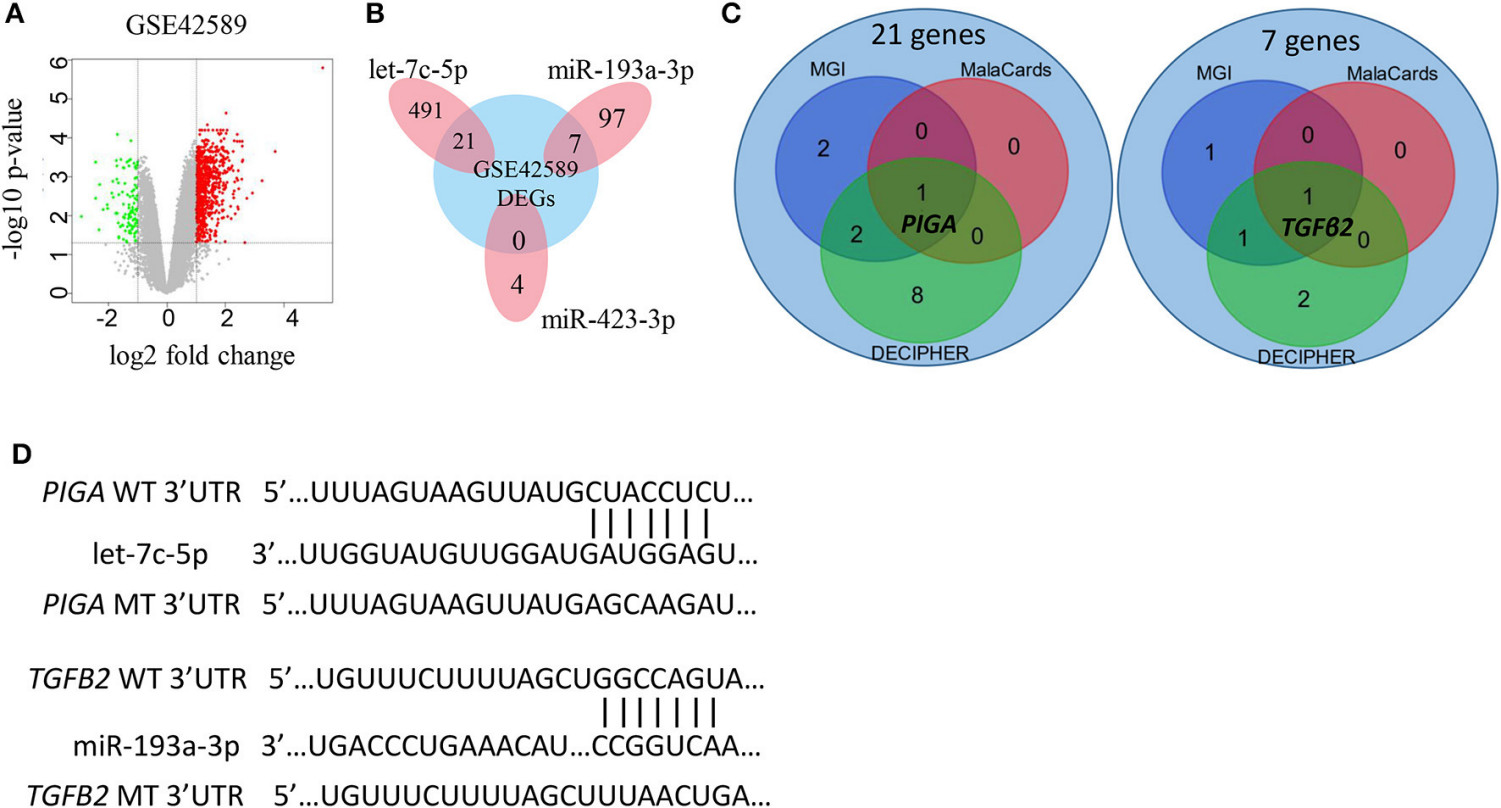
Selection of potential miRNA target genes. **(A)** Volcano plot of differentially expressed genes (DEGs) in GSE42589. **(B)** Target genes of the three miRNAs intersected with DEGs in GSE42589, respectively. A total of 28 genes are considered as susceptible genes for non-syndromic cleft lip with or without cleft palate. **(C)** Distribution of the 21 and 7 susceptible genes for non-syndromic cleft lip with or without cleft palate in three databases. MGI, Mouse Genome Informatics database; MalaCards, A Comprehensive Automatically-Mined Database of Human Diseases; DECIPHER, DatabasE of genomiC varIation and Phenotype in Humans using Ensembl Resources. **(D)** The predicted let-7c-5p and miR-193a-3p binding sites in the WT of 3′-UTR (untranslated region) of *PIGA* and *TGFB2*, as well as the MT of *PIGA* and *TGFB2*. WT, wide type; MT, mutation type.

Genes predicted in all three databases (FUNRICH, MIRDB, and Targetscan) were considered as potential target genes of the miRNAs. As a result, 104 target genes of miR-193a-5p, 512 target genes of let-7c-5p, and four target genes of miR-423-3p were initially identified (Supplementary Figure 2). These genes were then compared to the differentially expressed genes from NSCL/P cases and controls (GSE42589) (Figure 3A), and 21 genes targeted by let-7c-5p and seven genes targeted by miR-193a-3p were filtered out (Figure 3B). No target gene of miR-423-3p was identified. Finally, *PIGA* and *TGFB2* were selected as the most promising targets by further inquiring into three databases: MGI, MalaCards, and DECIPHER (Figure 3C). The binding sequence of miRNA-mRNA networks as well as the mutation of the 3'-UTR of *PIGA/TGFB2* are shown in Figure 3D.

### *PIGA* and *TGFB2* Are Target Genes of Let-7c-5p and miR-193a-3p

To prove that *PIGA* and *TGFB2* are target genes of let-7c-5p and miR-193a-3p, dual-luciferase reporter assays, qRT-PCR, and Western blotting were performed.

**Figure 4:**
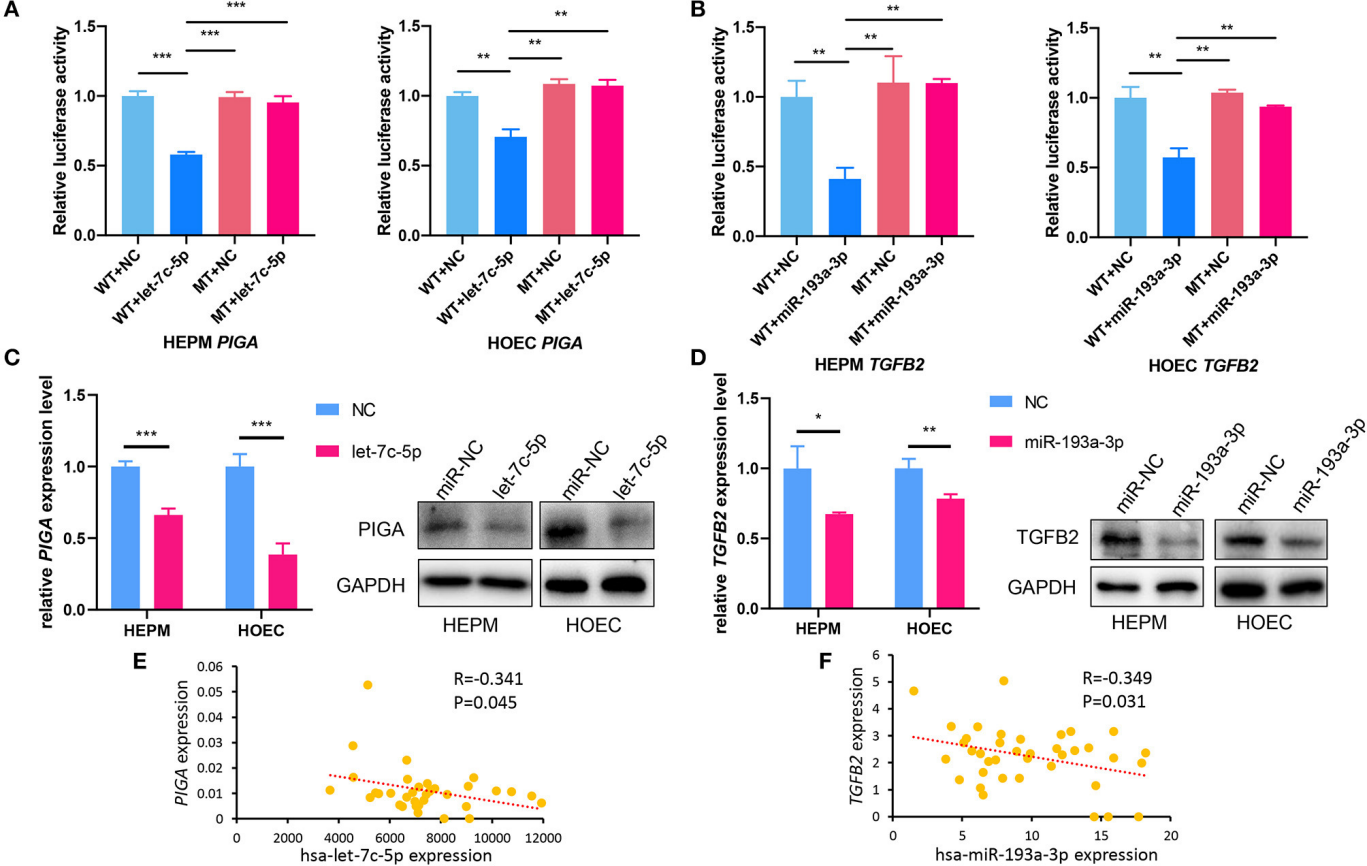
*PIGA* and *TGFB2* are target genes of let-7c-5p and miR-193a-3p, respectively. **(A)** Dual-luciferase reporter assays showed that overexpression of let-7c-5p **(A)** and miR-193a-3p **(B)** can decrease the luciferase activity of the WT 3′-UTR of *PIGA* and *TGFB2*, respectively, but did not influence the MT of 3′-UTR. **(C)** qPCR and Western Blot showed that overexpression of let-7c-5p **(C)** and miR-193a-3p **(D)** significantly reduced mRNA and protein expression of *PIGA* and *TGFB2*. **(E,F)** The inverse correlation between has-let-7c-5p-*PIGA* **(E)** and has-miR-193a-3p-*TGFB2* **(F)** was detected in NSCL/P lip tissue samples. WT, Wild-type; MT, Mutation-type; NC, Negative control; UTR, untranslated region, NSCL/P, non-syndromic cleft lip with or without cleft palate. *P < 0.05, **P < 0.01, ***P < 0.001.

Dual-luciferase reporter assays showed that the relative luciferase activities decreased in the 3'-UTR of *PIGA* and *TGFB2* compared with those in the control group when transfected with let-7c-5p and miR-193a-3p in HOECs and HEPM cells (Figures 4A,B). Nevertheless, there were no significant changes in the mutation types of *PIGA* and *TGFB2*. Likewise, qRT-PCR and western blot also showed that the mRNA and protein levels of *PIGA* (Figure 4C) and *TGFB2* (Figure 4D) decreased in cells transfected with let-7c-5p and miR-193a-3p mimics compared with those in the control group.

In addition, the expression of let-7c-5p, miR-193a-3p, *PIGA*, and *TGFB2* was detected in lip tissue samples from 40 NSCL/P patients who underwent surgery. A moderate negative correlation between hsa-let-7c-5p and *PIGA* (*r* = −0.341, *P* = 0.045) as well as between hsa-193a-3p and *TGFB2* (*r* = −0.349, *P* = 0.031) was observed by Spearman's correlation (Figures 4E,F).

Further, the association of rs77246858 in *TGFB2* with an increased risk of NSCL/P was detected in 504 NSCL/P cases and 455 control subjects (*P* = 4.88 × 10^−02^) (Supplementary Table 3). Rs77246858 is located in the vital regulatory elements of the gene, according to HaploReg v4 (Supplementary Figure 3A). 3D chromatin looping data showed that *TGFB2* interacted with rs77246858 in blood and skin (Supplementary Figures 3B,C), suggesting that it may modulate *TGFB2* through chromatin looping.

### Gene Expression in Mouse Craniofacial Development

RNA-Seq count data in the FaceBase repository for craniofacial research (www.facebase.org) revealed the expression of *piga* and *tgfb2* in E10.5–E14.5 mouse embryos, indicating their essential role in craniofacial development (Supplementary Figures 4A,B).

### Effects of Let-7c-5p and miR-193a-3p on Human Embryonic Palatal Mesenchyme Cells and Human Oral Epithelial Cells

**Figure 5:**
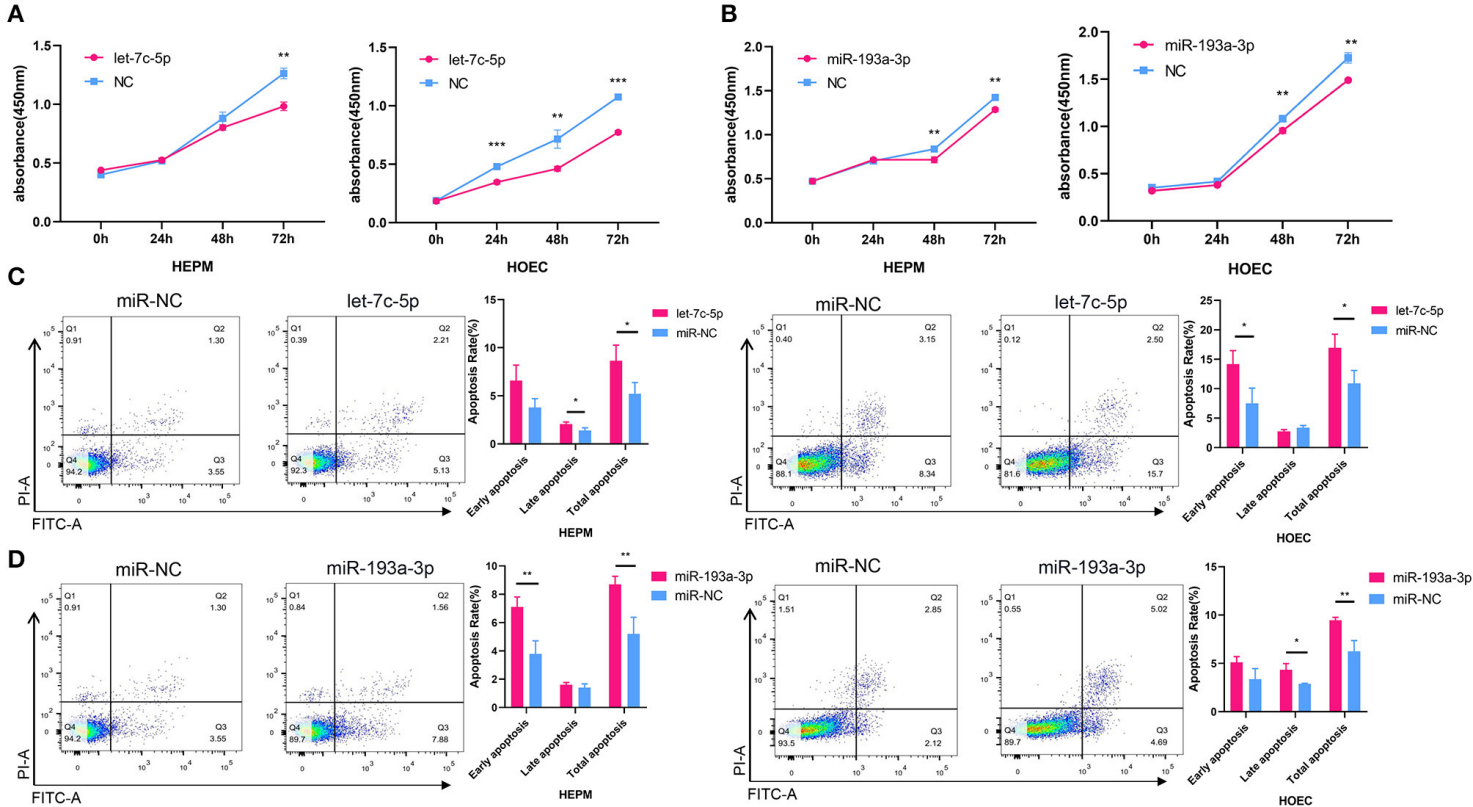
Effects of miRNA mimics on cell proliferation and apoptosis. **(A,B)** CCK-8 analysis showed let-7c-5p **(A)** and miR-193a-3p **(B)** can suppress the proliferation of HEPM and HOEC compared with NC. **(C,D)** Flow cytometric analysis was used to access the apoptosis rate of HEPM and HOEC after transfected with miRNA mimics. Let-7c-5p **(C)** and miR-193a-3p **(D)** promote apoptosis of HEPM and HOEC compared with NC. NC, Negative control; HEPM, Human embryonic palatal mesenchyme; HOEC, Human oral epithelial cells. *P < 0.05, **P < 0.01, ***P < 0.001.

To investigate the effects of let-7c-5p and miR-193a-3p on the proliferation and apoptosis of HEPM cells and HOECs, we transfected the two miRNA mimics and negative control into both cell lines. The CCK-8 assay showed that overexpression of let-7c-5p and miR-193a-3p inhibited the proliferation of HEPM cells and HOECs (Figures 5A,B). Correspondingly, flow cytometry analysis showed that the apoptosis rate of HEPM cells and HOECs was significantly increased compared with that of the negative control group when transfected with let-7c-5p and miR-193a-3p mimics (Figures 5C,D).

## Discussion

In the present study, we combined the DEMs during mouse embryonic palatal development with DEMs in NSCL/P patients, and identified a let-7c-5p-*PIGA* and miR-193a-3p-*TGFB2* network that may play important roles in the development of NSCL/P.

Let-7c-5p is a collection of miRNAs with high expression in the craniofacial tissues of embryonic mice. The let-7 miRNA family was reported to be extensively involved in regulation of cell proliferation and differentiation (Johnson et al., 2007). Here, we found that let-7c-5p overexpression promoted apoptosis and inhibited the proliferation of HEPM cells and HOECs. Iwata et al. previously found that decreased cell proliferation and increased cell death in cranial neural crest cells and lip mesenchymal cells can cause severe craniofacial anomalies. Given that these cells are derived from neural crest cells, we speculate that let-7c-5p may modulate craniofacial development through a similar mechanism (Suzuki et al., 2019).

*PIGA* was found to be a target of Let-7c-5p. *PIGA* can affect the development of the nervous system (Johnston et al., 2012). Johnston et al. reported that c.1234C>T in *PIGA* resulted in a lethal X-linked phenotype recognized in a family with an X-linked lethal disorder that involved cleft palate (CP), neonatal seizures, central nervous system (CNS) structural malformations, and other abnormalities. Meanwhile, mouse models in MGI suggested that *PIGA* mutation may lead to abnormal craniofacial bone morphology and cleft lip and palate phenotypes. In MalaCards, *PIGA* was found to be associated with an isolated CP. Among the DECIPHER database, three patients with rearrangement in the *PIGA* region of the chromosome had phenotypes such as abnormality of the mandible or CP.

MiR-193a-3p was also associated with the development of NSCL/P in the present study. MiR-193a-3p has been linked to a variety of tumor diseases, including oral cancer and cutaneous melanoma (Kozaki et al., 2008; Polini et al., 2020). Besides, plasma samples of patients with CP, and those with cleft lip with cleft palate, also showed low levels of miR-193a, which suggested that miRNA-193a-3p may be involved in the development of the oral cleft (Li et al., 2016).

Here, we found that *TGFB2* is a possible target gene of miR-193a-3p. *TGFB2* belongs to the transforming growth factor beta family, which regulates a wide variety of biological phenomena, such as cellular development, morphology, proliferation, apoptosis, and epithelial-mesenchymal transition (EMT), and has been considered to play an important role in the morphogenesis of the palate (Iordanskaia and Nawshad, 2011; Jin and Ding, 2014). Knockout of *TGFB2* in mice results in perinatal mortality and a wide range of developmental defects, including cardiac, lung, craniofacial, and limb defects (Azhar et al., 2009). The MGI database revealed that *tgfb2* was involved in multiple phenotypes associated with CL/P, including cleft secondary palate, abnormal craniofacial bone morphology, and failure of palatal shelf elevation. MalaCards showed that *TGFB2* is related to cleft lip/palate and oral cleft. According to the DECIPHER database, a patient with deletion in the region of the *TGFB2* gene showed congenital diseases, including CP, coarse facial features, intellectual disability, and ventricular septal defect. Moreover, an NSCL/P case and control cohort showed that rs77246858, which may regulate the function of *TGFB2*, is associated with the risk of NSCL/P.

In conclusion, our findings revealed that let-7c-5p and miR-193a-3p may play important roles in the development of NSCL/P by targeting *PIGA* and *TGFB2*, respectively. However, more experiments and functional studies are required to explore the causation between the miRNA-mRNA networks and the disease as well as the underlying mechanisms in the future.

## Data Availability Statement

The datasets presented in this study can be found in online repositories. The names of the repository/repositories and accession number(s) can be found at: https://www.ncbi.nlm.nih.gov/geo/, GSE47939, https://www.ncbi.nlm.nih.gov/geo/, GSE20880.

## Ethics Statement

The studies involving human participants were reviewed and approved by Institutional Review Board of Nanjing Medical University. Written informed consent to participate in this study was provided by the participants' legal guardian/next of kin.

## Author Contributions

CF analyzed the data, drafted the manuscript, and performed *in vivo* and *in vitro* experiments with SL. LM, YP, and LW designed and directed the study, obtained financial support, and critically revised the manuscript. GZ, LF, XY, and WZ performed the statistical analysis. All authors read and approved the final manuscript.

## Conflict of Interest

The authors declare that the research was conducted in the absence of any commercial or financial relationships that could be construed as a potential conflict of interest.
