# Supplementary material for: Identification of New miRNA-mRNA Networks in the Development of Non-syndromic Cleft Lip With or Without Cleft Palate
Source: Front Cell Dev Biol. 2021 Mar 1;9:631057. doi: 10.3389/fcell.2021.631057 (PMC7957012; doi:10.3389/fcell.2021.631057)
Supplement: Supplementary file 1 [file Table_1.DOCX]

| Supplementary Table 1. The information from selected databases | | | | | | |
| --- | --- | --- | --- | --- | --- | --- |
| Type | Database | Year | Organization | Platform | case | control |
| Human miRNA Microarray | GSE47939 | 2013 | Children Hospital of Fudan University | GPL11487 | 10 NSCP patients | 6 control |
| Embryonic murine miRNA Microarray | GSE20880 | 2019 | University of Louisville | GPL10179 | 3 murine embryonic orofacial tissues on gestational days (GD) 12 VS GD14 & GD13 VS GD14 & GD12 VS GD13 | |
| Human Gene Array | GSE42589 | 2018 | University of Sao Paulo | GPL6244 | 7 CL/P dental pulp stem cells RNA | 6 dental pulp stem cells RNA |

NSCP: non-syndromic cleft palate; GD: gestational days; NSCL/P: Non-syndromic cleft lip with or without palate.

| Supplementary Table 2. Primer sequences of *PIGA*, *TGFB2* and *GAPDH* | |
| --- | --- |
| Gene | Primer Sequences (5’-3’) |
| *PIGA* | F: GGTATATGACCGGGTATCAGTGG |
|  | R: GCAAAGATGTAGCCTGTTACTGG |
| *TGFB2* | F: CCATCCCGCCCACTTTCTAC |
|  | R: AGCTCAATCCGTTGTTCAGGC |
| *GAPDH* | F: ACAACTTTGGTATCGTGGAAGG |
|  | R: GCCATCACGCCACAGTTTC |
| F: Forward primer; R: Reversed primer. | |

| Supplementary Table 3. Demographic characteristic information of the NSCL/P cohort | | | | | |
| --- | --- | --- | --- | --- | --- |
|  | Case  (N=504) | |  | Control  (N=455^a^) | |
|  | N | % |  | N | % |
| Age (Mean±SD) | 1.51±0.51 | |  | 0.00±0.00 | |
| Gender, % |  |  |  |  |  |
| Male | 308 | 61.1 |  | 236 | 51.9 |
| Female | 196 | 38.9 |  | 219 | 48.1 |

NSCL/P, non-syndromic cleft lip with or without cleft palate; SD, standard deviation; a, newborn infant.

| Supplementary Table 4. LogFC and *P* values of DEMs selected from GSE47939 and GSE20880 | | | | | | |
| --- | --- | --- | --- | --- | --- | --- |
| ID | logFC | *P* value | ID | logFC | *P* value | Gestational days |
| **GSE47939** |  |  | **GSE20880** |  |  |  |
| hsa-let-7c-5p | 3.13 | 3.60E-02 | hsa-let-7b | -1.61 | 6.86E-04 | 12VS14 |
| hsa-miR-1306 | 2.99 | 1.90E-02 | hsa-let-7c-5p | 1.07 | 2.68E-03 | 12VS14 |
| hsa-miR-148b | 1.84 | 4.36E-02 | hsa-miR-101b | 1.06 | 4.05E-02 | 13VS14 |
| hsa-miR-181b | 2.74 | 2.23E-02 | hsa-miR-106a | 1.05 | 1.35E-02 | 12VS14 |
| hsa-miR-193a-3p | -4.26 | 2.21E-03 |  | 2.12 | 2.77E-02 | 13VS14 |
| hsa-miR-193a-5p | -4.35 | 5.22E-03 | hsa-miR-1224-3p | 1.50 | 1.77E-02 | 12VS13 |
| hsa-miR-23a | -3.37 | 3.26E-02 |  | 2.63 | 1.32E-02 | 12VS14 |
| hsa-miR-24-1 | -2.30 | 3.09E-02 | hsa-miR-125b-2 | -1.30 | 7.17E-03 | 12VS13 |
| hsa-miR-29b-1 | 2.43 | 2.87E-02 | hsa-miR-133a | -1.19 | 2.18E-02 | 13VS14 |
| hsa-miR-33b | -3.06 | 2.06E-02 | hsa-miR-152 | -1.28 | 1.84E-02 | 12VS14 |
| hsa-miR-370 | -2.54 | 4.17E-02 | hsa-miR-188-3p | 2.30 | 3.94E-02 | 12VS13 |
| hsa-miR-423-3p | 2.14 | 4.86E-02 |  | -3.09 | 2.33E-02 | 13VS14 |
| hsa-miR-484 | 1.99 | 4.49E-02 | hsa-miR-18a | 1.02 | 1.70E-02 | 12VS14 |
| hsa-miR-486-3p | 1.98 | 4.87E-02 | hsa-miR-18b | 1.00 | 2.66E-02 | 12VS14 |
| hsa-miR-498 | 2.41 | 2.32E-02 | hsa-miR-193a-3p | -2.85 | 2.71E-04 | 12VS13 |
| hsa-miR-499-5p | 2.10 | 3.86E-02 |  | -3.62 | 5.60E-05 | 12VS14 |
| hsa-miR-632 | 2.06 | 4.40E-02 | hsa-miR-193b | -2.18 | 2.79E-02 | 12VS14 |
| hsa-miR-654-3p | -2.35 | 2.80E-02 | hsa-miR-20a | 1.00 | 5.13E-03 | 12VS14 |
| hsa-miR-654-5p | -2.78 | 2.77E-02 | hsa-miR-22 | -1.06 | 3.41E-03 | 13VS14 |
| hsa-miR-664 | -4.49 | 1.93E-04 | hsa-miR-296-3p | 1.15 | 6.13E-03 | 12VS14 |
|  |  |  | hsa-miR-301a | 1.21 | 1.93E-02 | 12VS14 |
|  |  |  | hsa-miR-301b | 1.13 | 1.99E-03 | 12VS14 |
|  |  |  | hsa-miR-30e | 1.28 | 5.48E-03 | 12VS14 |
|  |  |  | hsa-miR-31 | -1.05 | 4.43E-02 | 12VS14 |
|  |  |  | hsa-miR-362-5p | 1.94 | 3.68E-03 | 12VS14 |
|  |  |  | hsa-miR-371-5p | 1.63 | 3.15E-02 | 12VS13 |
|  |  |  | hsa-miR-422a | -1.50 | 1.79E-02 | 12VS14 |
|  |  |  | hsa-miR-423-3p | 1.58 | 1.01E-02 | 12VS14 |
|  |  |  | hsa-miR-450a | -1.42 | 2.11E-02 | 12VS13 |
|  |  |  | hsa-miR-451 | -1.17 | 7.79E-03 | 12VS14 |
|  |  |  |  | -1.45 | 1.38E-02 | 13VS14 |
|  |  |  | hsa-miR-487b | -1.16 | 4.96E-02 | 12VS14 |
|  |  |  | hsa-miR-532-3p | 1.07 | 1.77E-02 | 12VS14 |
|  |  |  | hsa-miR-652 | 1.44 | 1.64E-02 | 12VS14 |
|  |  |  |  | 1.77 | 1.45E-02 | 13VS14 |
|  |  |  | hsa-miR-940 | 2.01 | 2.13E-02 | 12VS14 |
|  |  |  |  | 1.43 | 1.66E-02 | 13VS14 |

DEMs: differentially expressed miRNAs；logFC: log2 fold change；*P*-value < 0.05 was considered statistically significant.

| Supplementary Table 5. The association of rs77246858 with the risk of NSCL/P | | | | | | | | | | |
| --- | --- | --- | --- | --- | --- | --- | --- | --- | --- | --- |
| SNP | Chromosome | Allele | MAF | |  | Genotype | |  | *P* | OR (95% CI) |
|  | Position (hg19) | (Major>Minor) | Case | Control |  | Case | Control |  |  |  |
| rs77246858 | 1: 218599919 | T>C | 0.11 | 0.08 |  | 9/91/404 | 3/68/384 |  | 4.88 × 10^-02^ | 1.36(1.00–1.85) |

SNP, single-nucleotide polymorphism; NSCL/P, non-syndromic cleft lip with or without cleft palate; MAF, minor allele frequency; Genotype, shown as major/minor allele; OR, odds ratio; CI, confidence interval; *P*, *P* values were derived from logistic regression analysis under an additive model.


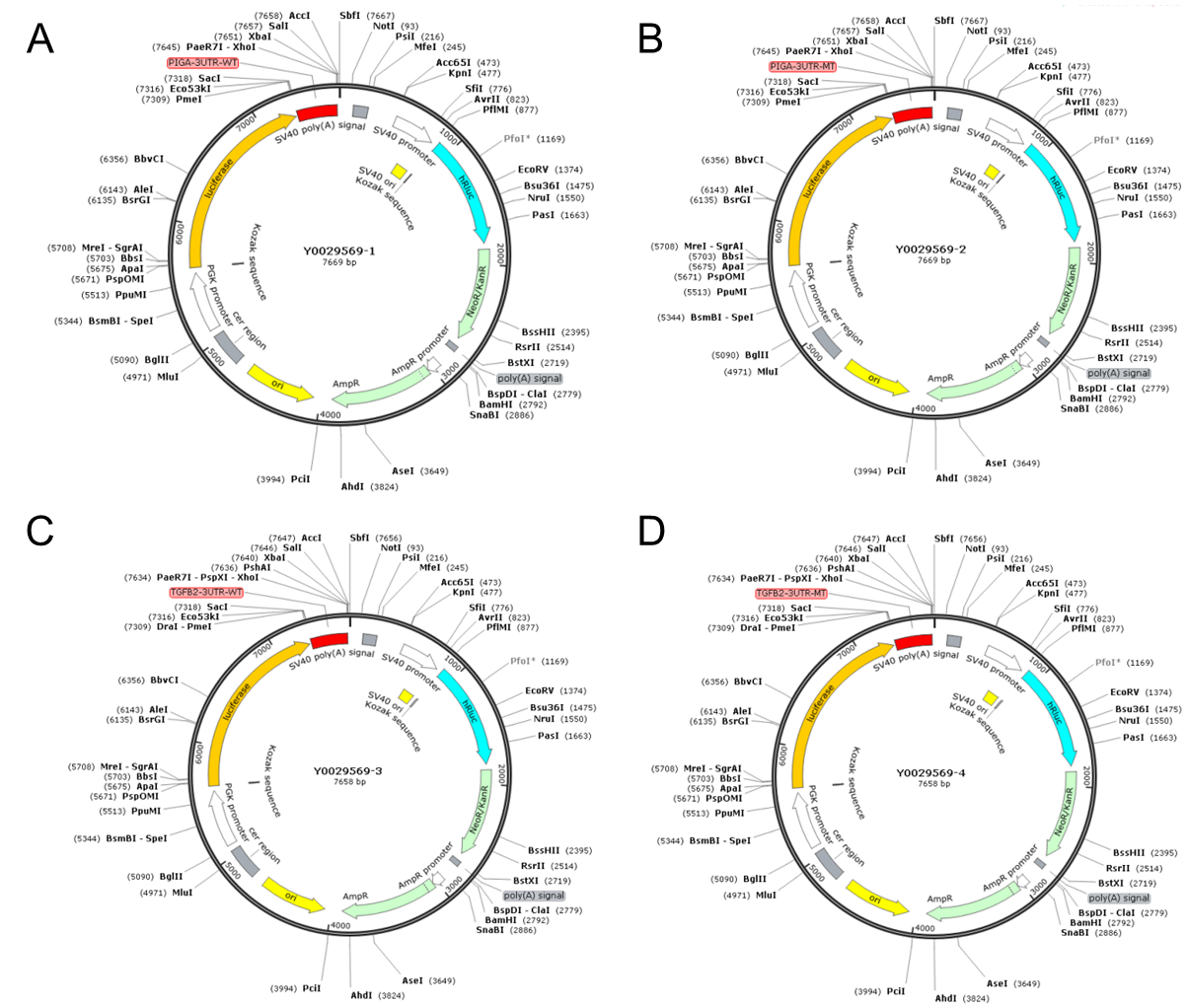


**Supplementary Figure 1.** Plasmid map of MT or WT of *PIGA* (A,B) and *TGFB2* (C,D). WT, wild type; MT, mutant type.


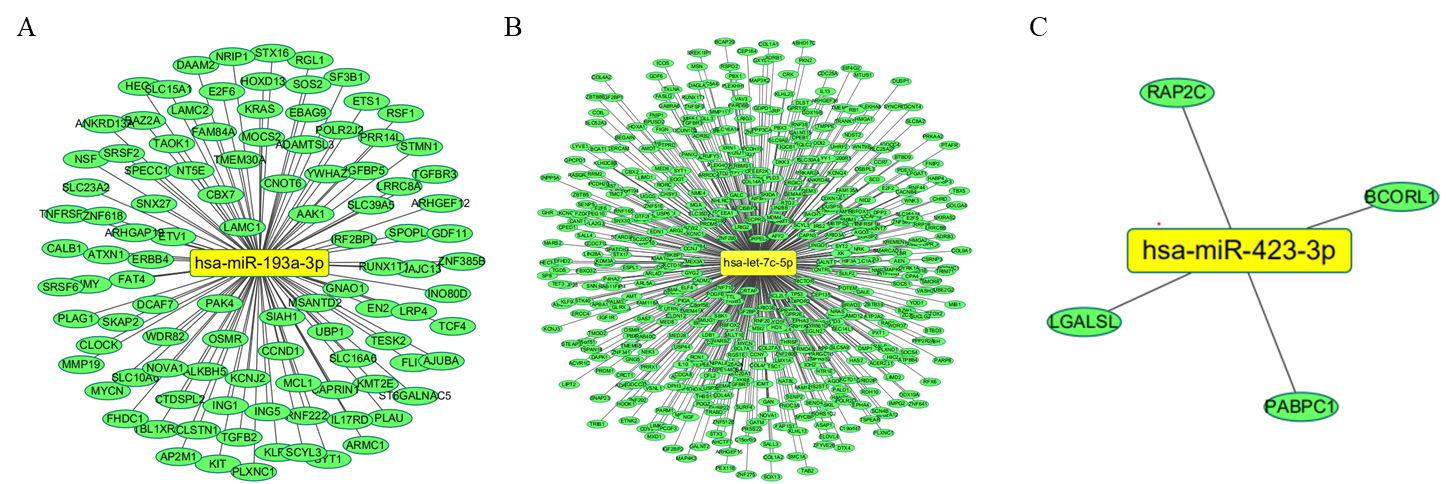


**Supplementary Figure 2.** Target genes of miR-193a-3p (A), let-7c-5p(B), and miR-423-3p(C) predicted by FUNRICH, MIRDB and Targetscan.


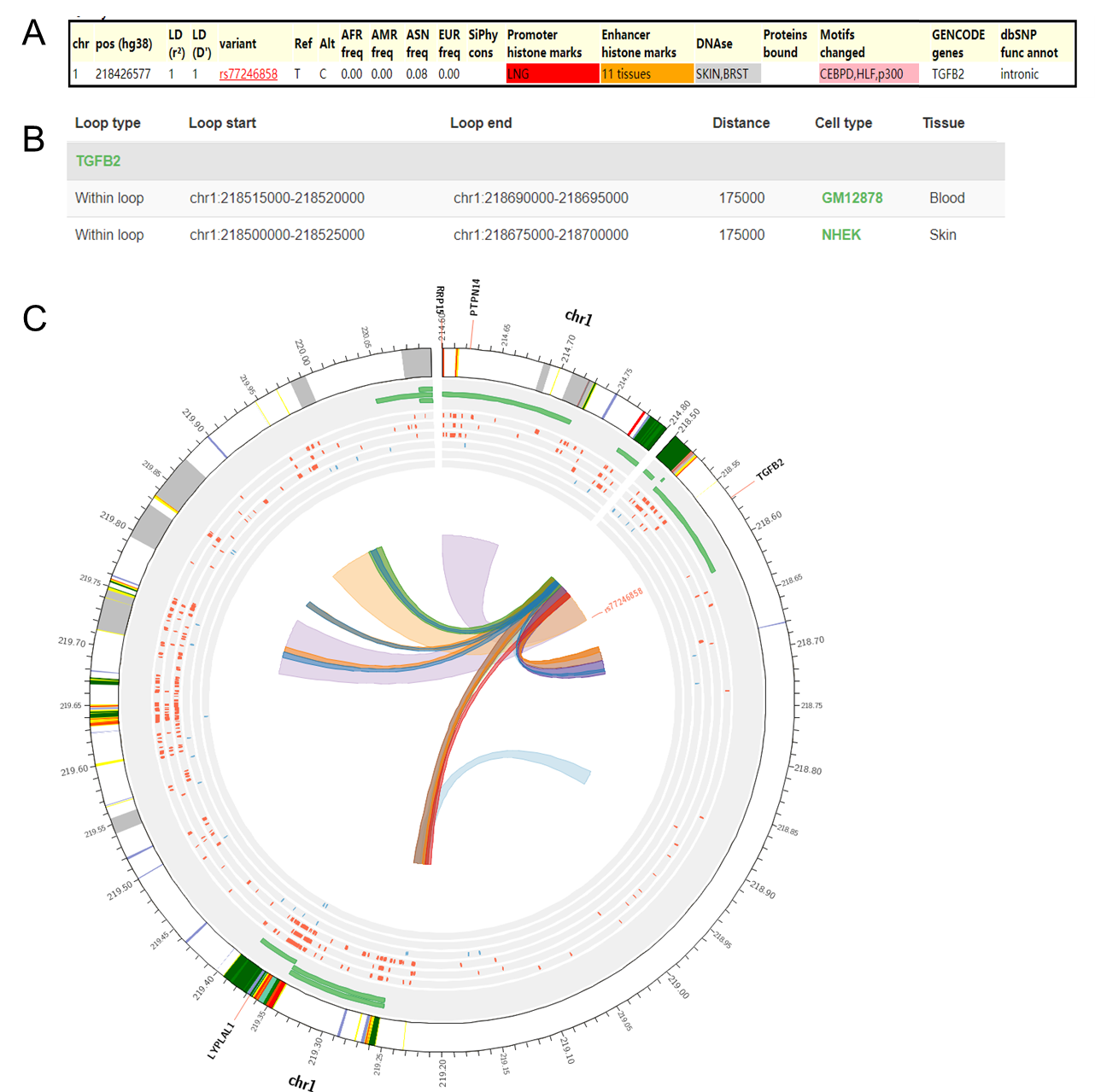


**Supplementary Figure 3.** Functional annotation of rs77246858. (A) HaploReg indicates that rs77246858 located in multiple regulatory regions of *TGFB2*. (B,C) 3DSNP (http://cbportal.org/3dsnp/) demonstrates that *TGFB2* is one of 3D interacting genes of rs77246858 within loop. There are three types of circles in the plots. The outer, middle, and inner circles represent ChromHMM chromatin states, annotated genes, and histone modification set (H3K4me1 and H3K4me3), respectively. In the circle are rs77246858 and the 3D interaction loops.


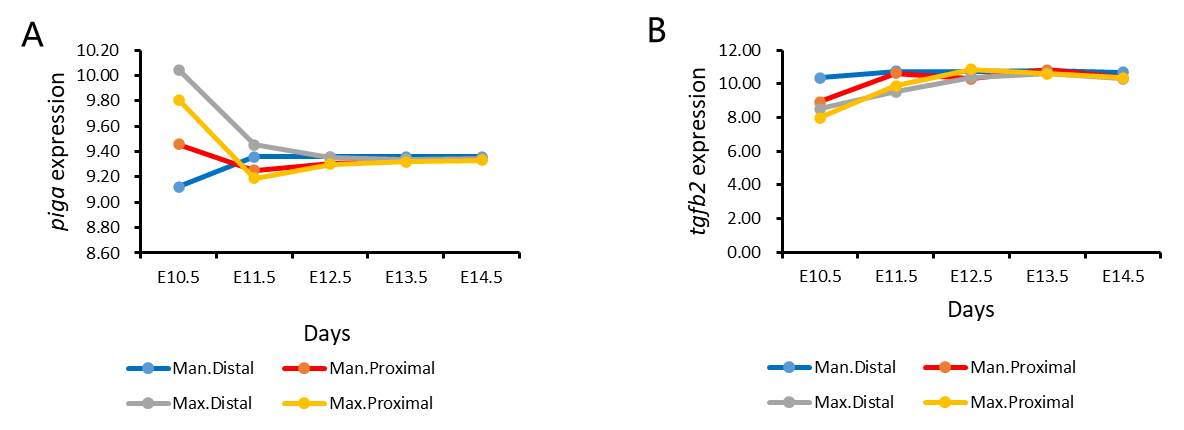


**Supplementary Figure 4.** Expression levels of *piga* (A) and *tgfb2* (B) in mouse craniofacial tissues during mouse embryo development stage from E10.5 days to E14.5 days according to Facebase database (http://www.facebase.org/, GSE67985). Man.Distal: Mandibular distal location; Man.Proximal: Mandibular proximal location; Max.Distal: Maxillary distal location; Max. Proximal: Maxillary proximal location.
